# Agricultural crop exposure and risk of childhood cancer: new findings from a case–control study in Spain

**DOI:** 10.1186/s12942-016-0047-7

**Published:** 2016-05-31

**Authors:** Diana Gómez-Barroso, Javier García-Pérez, Gonzalo López-Abente, Ibon Tamayo-Uria, Antonio Morales-Piga, Elena Pardo Romaguera, Rebeca Ramis

**Affiliations:** Consortium for Biomedical Research in Epidemiology and Public Health (CIBERESP), Madrid, Spain; National Center for Epidemiology, Carlos III Institute of Health, Monforte de Lemos 5, Pb. 12, 28029 Madrid, Spain; Cancer and Environmental Epidemiology Unit, National Center for Epidemiology, Carlos III Institute of Health, Madrid, Spain; Centre for Research in Environmental Epidemiology (CREAL), Barcelona, Spain; Universitat Pompeu Fabra (UPF), Barcelona, Spain; Rare Disease Research Institute (IIER), Carlos III Institute of Health, Madrid, Spain; Consortium for Biomedical Research in Rare Diseases (CIBERER), Madrid, Spain; Spanish Registry of Childhood Tumors (RETI-SEHOP), University of Valencia, Valencia, Spain

**Keywords:** Childhood cancer, Crops, Spatial epidemiology, GIS, Cases/control

## Abstract

**Background:**

Childhood cancer is the main cause of disease-related death in children in Spain. Although little is known about the etiology, environmental factors are potential explanations for a fraction of the cases. Previous studies have shown pesticides to be associated with childhood cancer. The difficulty of collecting personal environmental exposure data is an important limitation; this lack of information about pesticides motivates the development of new methods to subrogate this exposure. We developed a crop exposure index based on geographic information to study the relationship between exposure to different types of crops and risk of childhood tumors.

**Methods:**

We conducted a population-based case–control study of childhood cancer covering 3350 cases and 20,365 controls in two Spanish regions. We used CORINE Land Cover to obtain data about agricultural land use. We created a 1 km buffer around every child and calculated the percentage of crop surface within the buffer (Global Crop Index) for total crops and for individual types of crops. We fitted mixed multiple unconditional logistic regression models by diagnostic group.

**Results:**

We found excess of risk among children living in the proximity of crops. For total crops our results showed excesses of risk for almost all diagnostic groups and increasing risk with increasing crop index value. Analyses by region and individual type of crop also showed excess of risk.

**Conclusion:**

The results suggest that living in the proximity of cultivated land could be a risk factor for several types of cancer in children.

## Background

Childhood cancer is the main cause of disease-related death in childhood in Spain [1] and it is the leading cause of death among children aged 1–14 years [2]. The main group is leukemia, which has the highest incidence, (40 % of all cases) followed by tumors of the central nervous system (20 % of all cases) and lymphomas (15 % of all cases) [3]. Although little is known about the etiology of childhood cancer, environmental factors are potential explanations for a fraction of the incidence in the different diagnostic groups. Research about the influence of environmental factors on childhood cancer genesis has primarily focused on parental occupational exposures and less on the direct exposure of the children. However, regarding exposure to pesticides, a number of epidemiological studies have shown association with risk of childhood cancer [1, 4–8]; potential mechanisms by which pesticide exposure may lead to cancer in children still remain speculative. This existing literature shows that there is some evidence of association between pesticide exposure and childhood leukemia, and little evidence of association for other cancer subtypes such as brain cancer, neuroblastoma, non-Hodgkin’s lymphoma or Wilms’ tumor [7, 9–11].

Agricultural exposures may encompass a variety of chemical and physical agents, but pesticides are usually of the greatest interest. Pesticides are biologically active molecules that are commonly used to destroy unwanted organisms in agricultural and residential environments. As a group, agricultural pesticides include herbicides, insecticides, fungicides, rodenticides, and other biocides [12]. The difficulty of collecting data about this type of exposure has encouraged the use of different methodologies to approximate it. A number of studies have used Geographic Information Systems (GIS) and remote sensing technologies to aid in agricultural pesticide exposure research [13–18]. Many of these methods incorporate spatial functions such as distance measurement, buffering, and overlay analysis. One of these studies showed a method to create historical crop maps using a GIS to determine whether crop maps are useful for predicting levels of crop herbicides in carpet dust samples from residences [16]. Another study used these tools for assessing the association between different crop patterns around the mothers’ residences and the birth weight of babies [17]. These approximations on individual exposure to pesticides may have some limitations [19] but in many cases they are the only way to approach the problem and to highlight environmental hazards.

The lack of data about individual exposure to pesticides in the study of childhood cancer has motivated the development of a method to subrogate this exposure using land use data from the European Environment Agency. The objectives of this paper are twofold: to show how we constructed this index, so it can be replicated in other countries or regions; and to study the potential association with cancer in children in a population-based case–control study.

## Methods

### Data

The results presented in this paper come from a research project which studied environmental risk factors for childhood cancer in Spain using the geographic locations of the cases and controls. The design of the study is a population-based case–control study and specific details can be found in previous papers from the project [20, 21]. For the reader’s convenience, there is a summary of the design below.

**Fig. 1:**
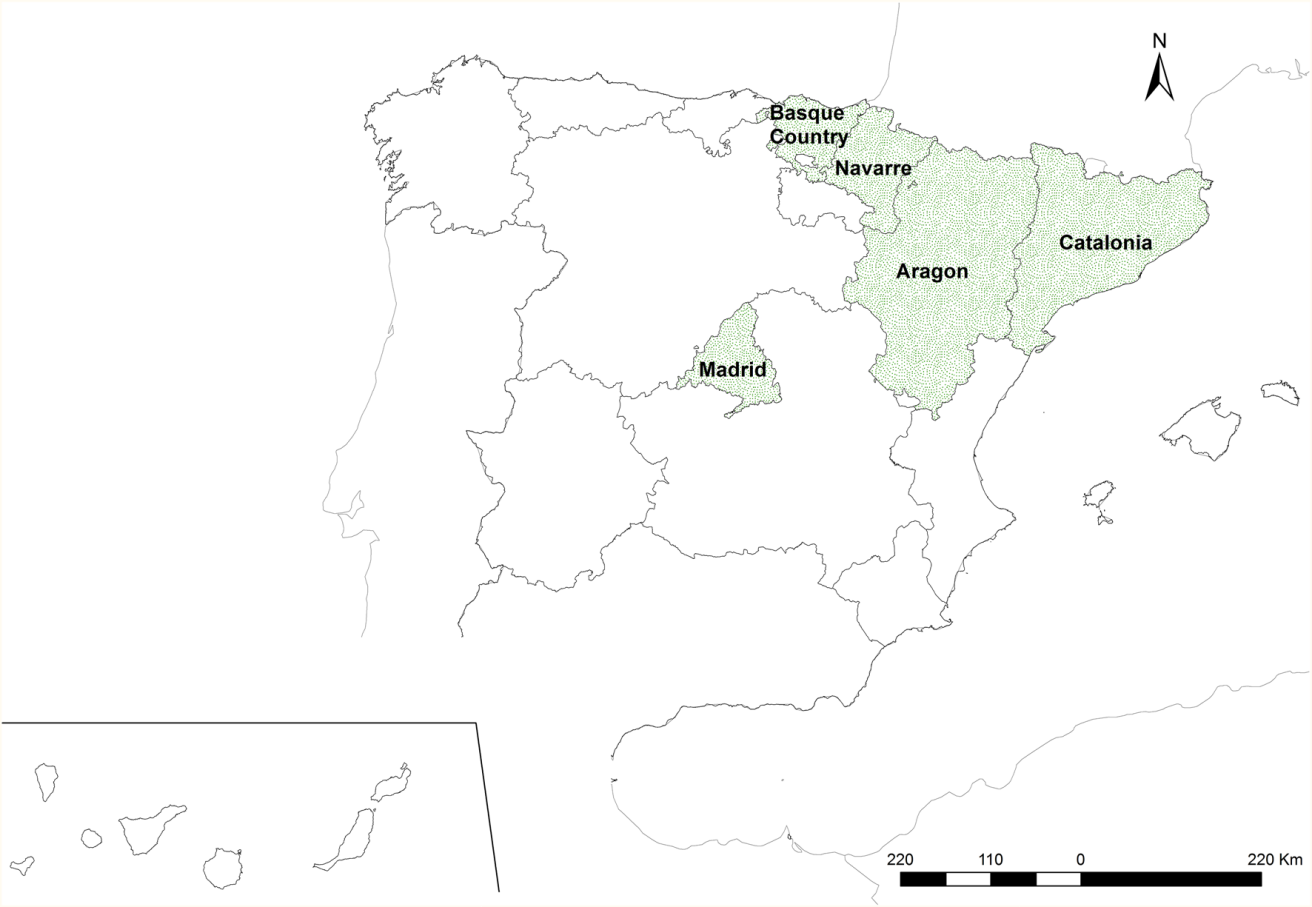
Spanish regions that participated in the study

The data used for the study were from children aged 0–14 with a diagnosed cancer such as leukemia, lymphomas (Hodgkin lymphoma: HL, and non-Hodgkin lymphoma: NHL), central nervous system neoplasms (CNS), neuroblastoma, retinoblastoma, renal tumors, hepatic tumors, malignant bone tumors, soft tissue and extra osseous sarcomas or germ cell tumors, groups I to X from the International Classification of Childhood Cancer, Third Edition (ICCC-3) [22]. The incident cases were registered by the Spanish Registry of Childhood Tumors (RETI-SEHOP), run by the Spanish Society of Pediatric Hematology and Oncology. RETI-SEHOP collects information from cases of childhood cancer from the regional cancer registries and pediatric oncology units all over Spain [3]. The completeness of the national coverage of childhood cancer by this registry is estimated at over 90 %, and at 100 % for the following five regions: Catalonia, Aragon, Navarre, the Basque Country and the Autonomous Region of Madrid. The studied period went from year 1996 to year 2011. As a control group, we used a sample from the population at risk extracted from the Birth Registry of the National Statistics Institute (Instituto Nacional de Estadística, INE). The cases were matched by year of birth, autonomous region of residence and sex, with six controls. For the cases, we geocoded the address at diagnosis, address included in the register RETI-SHEOP, and for the controls we used the mother’s address from the Birth Register. We analyzed two disconnected Spanish areas: the Autonomous Region of Madrid (“Madrid region”), and a region in the north-east of the country that included the Autonomous Regions of the Basque country, Aragon, Navarre and Catalonia (“North region”). Figure 1 shows the exact location of these regions within Spain.

We geocoded and validated the addresses of the cases. We successfully validated 87 % of the addresses. The remaining 13 % of cases were fairly uniformly distributed through the different regions and therefore we concluded the data were not biased in this sense. We then geocoded the addresses of the controls and we validated the coordinates. Only 2 % of the controls did not have valid coordinates. Having had a very small number of failures, we decided to select more controls to replace this 2 %, and we geocoded and validated this last group to end up with six controls with valid coordinates for every case.

**Fig. 2:**
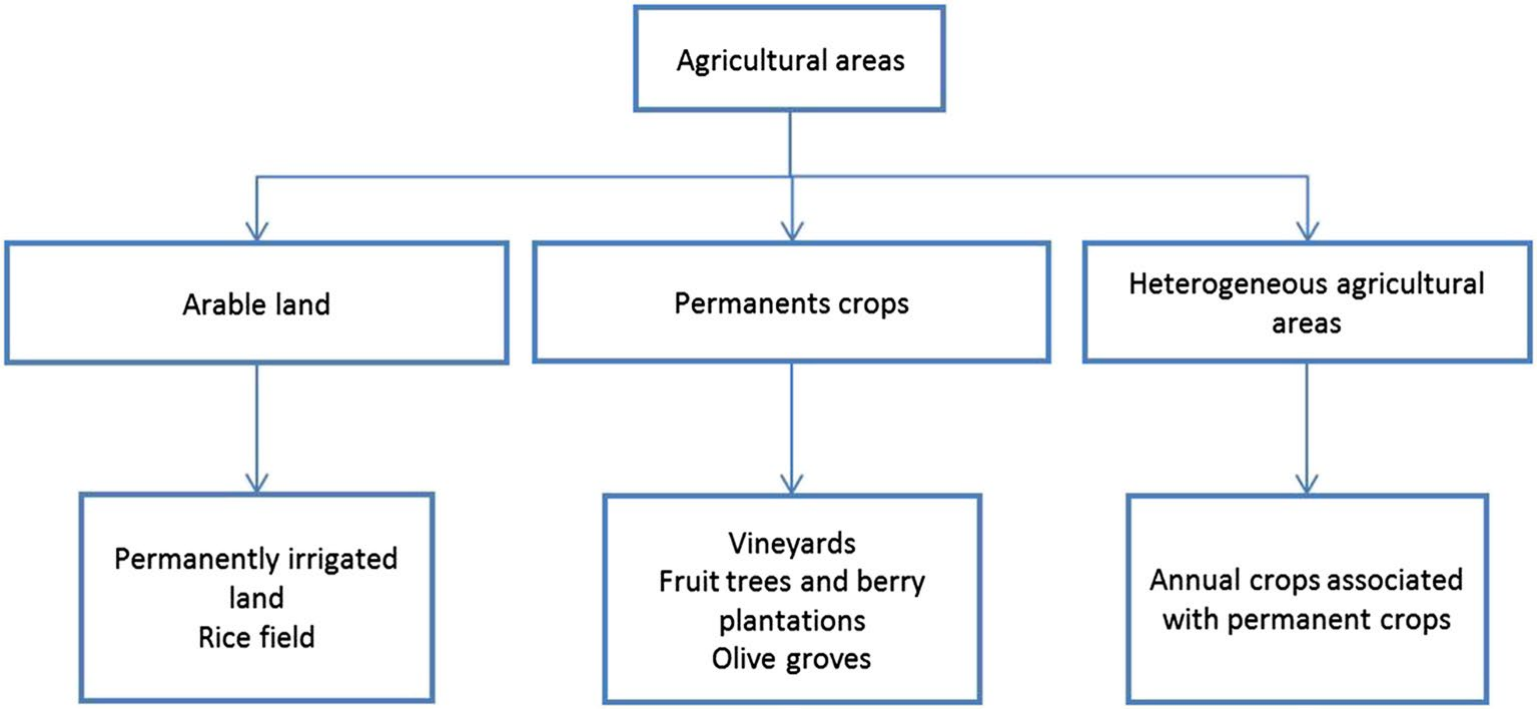
Agricultural areas

Because of the lack of data on exposure to pesticides and about the specific pesticides that were used on the Spanish crop fields, we decided to estimate the individual exposure to crop-associated factors by the definition of a new index. We used the CORINE Land Cover 2006 inventory from the European Environment Agency to obtain data about land use [23]. CORINE Land Cover is a spatial database with information about land use and land cover with a scale of 1:100,000. The land use is divided into five classes, one of which is “Agricultural areas”. The cartography covers most areas of Europe and the minimum size of the polygons is 25 ha. To build the index we chose all the polygons with this land use and, after that, we created a 1 km buffer around every child: case or control. We calculated the percentage of crop surface within every buffer and we designated this percentage as the “Global Crop Index”. The “Agricultural Areas” class is subdivided into six subcategories: arable land or permanently irrigated land (Irrigated); rice fields (Rice); vineyards (Vineyards); fruit trees and berry plantations (Fruits); olive groves (Olives) and heterogeneous agricultural areas, including annual crops associated with permanent crops (Heterogeneous) [23]. We also calculated the index for every individual subcategory. Figure 2 shows the surface for the “Agricultural areas” class and the subcategories within the studied regions. We defined as “exposed to crops” those children—cases or controls—with more than 0 % in the Global Crop Index.

In a second stage, we categorized the indexes to allow non-linear relationships between the increment of percentage and the risk of cancer. To build these categorized variables, we first removed the non-exposed individuals and then we computed the quartiles of the variable for the exposed individuals. We repeated this process for every type of crop. Finally, we assigned every child to the equivalent category ending up with a variable of five categories: 0 (Not exposed), 1 (1st quartile), 2 (2nd quartile), 3 (3rd quartile) and 4 (4th quartile). We computed the categorized variables for the North region and Madrid region separately.

To include information about potential confounders in the model, we also collected data about exposure to industrial pollution and about socio-economic status. For the exposure to industrial pollution we used the industrial database (industries governed by IPPC and facilities pertaining to industrial activities not subject to IPPC but included in the E-PRTR) provided by the Spanish Ministry for Agriculture, Food & Environment in 2009, which includes information on the geographic location and industrial pollution emissions of all industrial plants in Spain. We defined a 2.5 km buffer around the industry to calculated cases and control exposure [20]. For the data about socio-economic status we did not have individualized information so we decided to use the data from the 2001 Census [24]. This census has information at census tract level so we assigned the information of the corresponding census tract to every child. We selected data for unemployment and socio-economic level.

### Statistical analysis

In order to estimate the Odds Ratio and 95 % confidence intervals (95 % CIs) associated with the Global Crop Index and individual indexes we fitted mixed multiple unconditional logistic regression models for the North regions, including the region as random effect, and we fitted multiple unconditional logistic regression models for Madrid region. In both cases we adjusted the models by socio-economic covariates (unemployment and socio-economic condition) and for industrial pollution exposure. We only computed the analysis with those cancers with three or more cases in the exposure category. We also fitted the same models using the categorized variables and then we computed trend tests to evaluate the potential increase in the ORs. We used R library Lmer4 [25] for statistical analysis and ArcGIS 10.0 to build the indexes.

## Results

After the geocoding and validation of postal addresses, we ended up with 3350 cases (1062 of leukemia, 92 HL, 245 of NHL, 711 of CNS, 398 of neuroblastoma, 139 of retinoblastoma, 212 of renal tumors, 57 of hepatic tumors, 114 of malignant bone tumors, 200 of sarcomas and 120 of germ cell tumors) and 20,365 controls. We performed separate analyses for the North regions and Madrid region. Table 1 shows a disaggregation of cases by cancer subtype and administrative region, the mean age at diagnosis in years and sex ratio male/female.

**Table 1:** Number of cases by cancer subtype and administrative region, mean diagnostic age in months and sex ratio (M/F)

Diagnostic group	Aragon	Catalonia	Navarre	Basque country	North regions	Madrid region	Mean diagnostic age (years)	Sex ratio (M/F)
Leukemia	60	418	41	119	638	424	4.5	1.4
HL	6	35	3	9	53	39	8.7	2.8
NHL	19	100	7	17	142	102	6.4	2.7
CNS	52	341	35	82	510	201	5	1.16
Neuroblastoma	16	176	15	50	257	141	1.7	1
Retinoblastoma	11	58	9	10	88	51	1.5	0.96
Renal tumors	18	94	11	21	144	68	2.7	0.9
Hepatic tumors	5	22	1	7	35	23	2.7	1.9
Malignant bone tumors	13	55	4	8	80	34	8.5	1.03
Sarcomas	10	85	11	25	131	69	4.6	1.6
Germ cell tumors	10	55	5	6	76	44	4.2	1.04
Total cases	220	1439	142	354	2154	1196	4.4	1.28
Controls	1295	8865	857	2133	13,150	7215	–	–

**Fig. 3:**
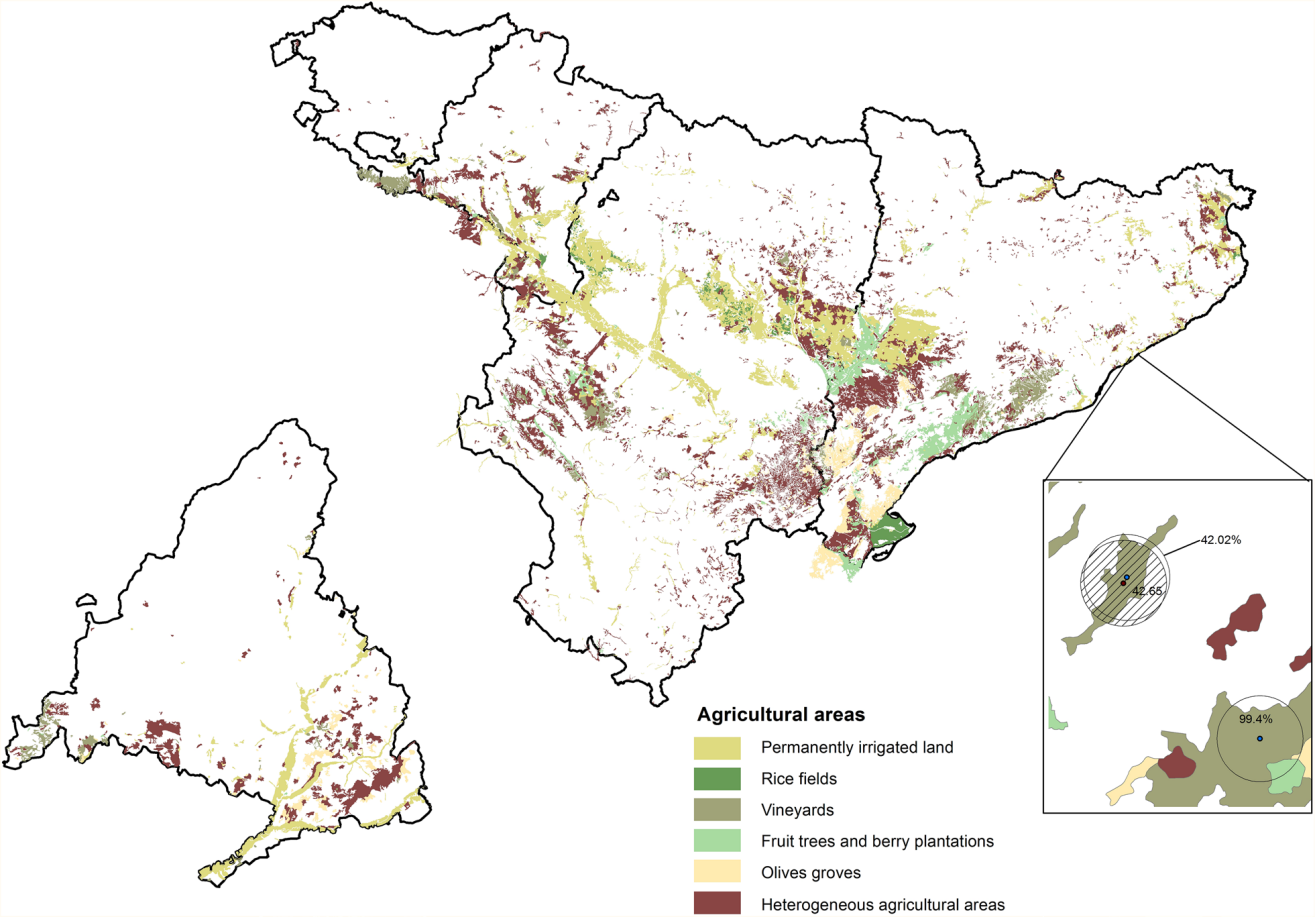
Spatial distribution of crops in “Madrid region” and “North region”

Table 2 shows the number of cases and controls exposed to crops. For all the diagnostic groups but retinoblastoma, the percentage of children exposed is two times or higher than the percentage of controls exposed. The exposure area is divided in a 38.4 % for irrigated, 33.2 % for heterogeneous, 15.6 % for fruit, 8.8 % for vineyards and 3.1 % for olives. Figure 3 shows the distribution of the areas of crops for the North and Madrid regions.

**Table 2:** Number of cases and controls exposed to crops

Diagnostic group	Total cases	Total controls	Exposed cases	Exposed controls	% Exposed cases	% Exposed controls
Leukemia	1062	6451	238	823	0.22	0.13
HL	92	552	21	94	0.23	0.17
NHL	246	1474	66	208	0.27	0.14
CNS	711	4255	186	587	0.26	0.14
Neuroblastoma	398	2375	101	298	0.25	0.13
Retinoblastoma	139	829	21	101	0.15	0.12
Renal tumors	212	1275	53	154	0.25	0.12
Hepatic tumors	58	350	17	41	0.29	0.12
Bone	114	667	26	121	0.23	0.18
Sarcomas	200	1196	50	131	0.25	0.11
Germ cell tumors	120	714	33	95	0.28	0.13
Total	3353	20,138	812	2653	0.24	0.13

We performed the analysis for the “Global Crop Index” and for every type of crop but rice because there were only a few children exposed to it. The results of the logistic regression models for the categorical variables are shown in Table 3. For the North regions and Global Crop Index the majority of the estimated ORs for the different categories and cancer subtype were above 1 and most of them were statistically significant. We observed increases in the ORs with increases in the quartiles for all cancer subtypes but retinoblastoma, and these trends were also statistically significant. NHL showed the highest ORs increasing from 2.12 for the first quartile to 5.59 for the last quartile; for leukemia the ORs increased from 1.76 to 2.57; and for CNS the ORs increased from 1.36 to 3.65. For the individual type of crops estimated OR’s behaved in a similar way to irrigated and heterogeneous crops with the main cancer subtypes, leukemia, NHL, CNS and neuroblastoma, showing positive trends. Only cases of the main cancer subtypes were exposed to fruits, vineyards and olives crops and here the results also showed increasing trends. CNS tumors showed a positive trend for all crop types. Madrid region had fewer types of crops; therefore, we performed the analysis with the categorical variable only for Crop Global Index. Table 4 shows the results and here again the main cancer subtypes showed positive trends except from CNS tumors.

**Table 3:** ORs and 95 % CI by quartiles with reference group: 0 % in the Global Crop Index

Crop Global Index	1Q (0–2.55]	2Q (2.55–8.91]	3Q (8.91–26.42]	4Q (26.42–100]	Trend PV
Leukemia	*1.76* (*1.26*, *2.48*)	*2.66* (*1.98*, *3.56*)	*2.83* (*2.08*, *3.85*)	*2.53* (*1.81*, *3.51*)	≤*0.05*
HL	1.07 (0.26, 4.49)	*2.78* (*1.07*, *7.18*)	*3.21* (*1.23*, *8.35*)	*2.74* (*0.92*, *8.15*)	≤*0.05*
NHL	*2.12* (*1.09*, *4.11*)	*2.17* (*1.12*, *4.23*)	*3.32* (*1.82*, *6.04*)	*5.6* (*3.30*, *9.50*)	≤*0.05*
CNS	1.36 (0.90, 2.06)	*2.05* (*1.44*, *2.92*)	*3.36* (*2.46*, *4.59*)	*3.65* (*2.66*, *5.01*)	≤*0.05*
Neuroblastoma	*1.84* (*1.09*, *3.12*)	*1.99* (*1.19*, *3.32*)	*3.92* (*2.55*, *6.01*)	*3.56* (*2.24*, *5.64*)	≤*0.05*
Retinoblastoma	1.52 (0.65, 3.53)	0.25 (0.03, 1.84)	1.46 (0.58, 3.68)	1.41 (0.54, 3.68)	
Renal tumors	1.72 (0.86, 3.44)	*1.82* (*0.91*, *3.65*)	*3.43* (*1.93*, *6.13*)	*3.24* (*1.71*, *6.17*)	≤*0.05*
Hepatic tumors		1.79 (0.41, 7.78)	*8.24* (*3.48*, *19.47*)	*3.93* (*1.22*, *12.61*)	≤*0.05*
Malignant bone tumors	1.05 (0.33, 3.38)	1.11 (0.34, 3.59)	*3.57* (*1.67*, *7.66*)	*4.91* (*2.36*, *10.22*)	≤*0.05*
Sarcomas	1.44 (0.66, 3.14)	1.68 (0.80, 3.50)	*2.68* (*1.41*, *5.12*)	*3.88* (*2.15*, *6.99*)	≤*0.05*
Germ cell tumors	2.01 (0.79, 5.10)	3.24 (1.51, 6.97)	*2.78* (*1.16*, *6.61*)	*4.72* (*2.23*, *9.96*)	≤*0.05*
Irrigated	1Q (0–2.23]	2Q (2.23–7.00]	3Q (7.00–19.10]	4Q (19.10–100]	Trend PV
Leukemia	*1.7* (*1.13*, *2.58*)	*3.10* (*2.19*, *4.42*)	*1.90* (*1.24*, *2.94*)	*2.13* (*1.39*, *3.30*)	≤*0.05*
HL	0.82 (0.12, 5.95)	2.03 (0.50, 8.41)		*3.57* (*1.09*, *12.04*)	
NHL	1.59 (0.65, 3.92)	*2.33* (*1.03*, *5.34*)	*3.9* (*1.03*, *7.57*)	*3.4* (*1.62*, *7.24*)	≤*0.05*
CNS	1.02 (0.58, 1.83)	*2.29* (*1.48*, *3.57*)	*2.19* (*1.40*, *3.45*)	*3.70*(*2.53*, *5.46*)	≤*0.05*
Neuroblastoma	1.45 (0.72, 2.98)	*3.12* (*1.79*, *5.45*)	*2.74* (*1.51*, *5.04*)	*3.15* (*1.74*, *5.77*)	≤*0.05*
Retinoblastoma	2.38 (0.97, 5.94)	0.58 (0.08, 4.21)	1.18 (0.29, 4.84)	2.60 (0.93, 7.39)	
Renal tumors	1.48 (0.61, 3.65)	0.72 (0.18, 2.94)	1.52 (0.57, 4.15)	*3.66* (*1.75*, *7.76*)	≤*0.05*
Hepatic tumors		1.74 (0.24, 12.89)	*6.71* (*2.33*, *19.70*)	*7.14* (*2.35*, *22.22*)	≤*0.05*
Malignant bone tumors	2.32 (0.86, 6.42)	*3.58* (*1.45*, *8.99*)	1.46 (0.37,6.04)	*3.5* (*1.25*,*9.99*)	≤*0.05*
Sarcomas	2.08 (0.92,4.77)	2.11 (0.87,5.23)	3.37 (1.64,7.02)	3.63 (1.72,7.76)	≤*0.05*
Germ cell tumors	0.62 (0.09,4.51)	*3.81* (*1.54*,*9.60*)	*4.49* (*1.94*,*10.58*)	*4.08* (*1.59*,*10.65*)	≤*0.05*
Heterogeneous	1Q (0–2.23]	2Q (2.23–6.52]	3Q (6.52–18.38]	4Q (18.38–100]	Trend PV
Leukemia	*1.31* (*0.79*,*2.20*)	*1.89* (*1.21*,*2.95*)	*2.94* (*1.98*,*4.37*)	*1.94* (*1.23*,*3.06*)	≤*0.05*
HL		3.11 (0.95,10.20)	1.2 (0.16,8.88)	*2.25* (*0.52*,*9.61*)	
NHL	1.53 (0.56,4.18)	*1.95* (*0.79*,*4.84*)	*1.35* (*0.42*,*4.32*)	*4.52* (*2.34*,*8.74*)	≤*0.05*
CNS	1.43 (0.83, 2.48)	*1.91* (*1.17*, *3.11*)	*2.79* (*1.79*, *4.36*)	*1.86* (*1.11*, *3.10*)	≤*0.05*
Neuroblastoma	0.83 (0.31, 2.26)	*1.73* (*0.84*, *3.57*)	*3.48* (*1.97*, *6.18*)	*2.47* (*1.30*, *4.69*)	≤*0.05*
Retinoblastoma	0.55 (0.08, 3.98)	0.56 (0.08, 4.07)	0.64 (0.09, 4.70)	1.75 (0.53, 5.71)	
Renal tumors	0.41 (0.06, 2.95)	2.63 (1.14, 6.08)	4.77 (2.36, 9.65)	*3.03* (*1.29*, *7.09*)	≤*0.05*
Hepatic tumors		1.38 (0.18, 10.27)	*1.49* (*0.20*, *11.23*)	*2.54* (*0.57*, *11.36*)	
Malignant bone tumors		*2.28* (*0.71*, *7.38*)	3.6 (1.28, 10.14)	*3.4* (*1.19*, *9.69*)	≤*0.05*
Sarcomas	0.76 (0.19, 3.11)	1.59 (0.58, 4.36)	1.79 (0.65, 4.97)	2.40 (1.02, 5.64)	≤*0.05*
Germ cell tumors	1.31 (0.32, 5.42)		*2.95* (*1.05*, *8.31*)	*1.97* (*0.60*, *6.49*)	
Fruits	1Q (0–1.91]	2Q (1.91–6.36]	3Q (6.36–19.42]	4Q (19.42–100]	Trend PV
Leukemia	*1.96* (*0.38*, *2.34*)	*2.23* (*0.41*, *2.15*)	*5.78* (*0.46*, *2.43*)	*2.98* (*0.83*, *3.32*)	
NHL	3.92 (1.41, 10.89)	4.11 (1.64, 10.31)	4.64 (1.84, 11.70)	*4.00* (*1.41*, *11.34*)	≤*0.05*
CNS	1.28 (0.50, 3.05)	*1.85* (*0.93*, *3.68*)	*2.55* (*1.36*, *4.80*)	*2.94* (*1.59*, *5.43*)	≤*0.05*
Neuroblastoma	2.36 (0.95, 5.85)	*1.59* (*0.57*, *4.27*)	*1.79* (*0.65*, *4.92*)	*3.74* (*1.76*, *7.92*)	≤*0.05*
Malignant bone tumors	3.59 (0.86, 14.96)	*2.99* (*0.72*, *12.47*)		*6.12* (*1.82*, *20.63*)	
Germ cell tumors	1.78 (0.24, 13.01)	3.02 (0.72, 12.63)	1.6 (0.22, 11.79)	3.32 (0.77, 14.21)	
Vineyards	1Q (0–2.23]	2Q (2.23–8.28]	3Q (8.28–22.60]	4Q (22.60–100]	Trend PV
Leukemia	0.74 (0.18, 3.03)	*2.08* (*0.95*, *4.55*)	*2.96* (*1.33*, *6.57*)	*0.98* (*0.30*, *3.13*)	≤*0.05*
SNC	0.93 (0.28, 3.83)	1.12 (0.35, 3.57)	3.7 (1.66, 8.23)	2.46 (1.05, 5.72)	≤*0.05*
Neuroblastoma	1.81 (0.44, 7.46)	*1.46* (*0.35*, *5.99*)	*3.06* (*0.94*, *9.94*)	*1.59* (*0.38*, *6.56*)	
Germ cell tumors	6.49 (1.54, 27.39)	2.55 (0.35, 18.77)	3.61 (0.49, 26.69)	2.73 (0.37, 20.22)	
Olives	1Q (0–1.23]	2Q (1.23–5.73]	3Q (5.73–12.73]	4Q (12.73–100]	Trend PV
Leukemia		*1.97* (*0.46*, *8.43*)	*2.85* (*0.84*, *9.63*)	*1.76* (*0.41*, *7.49*)	
LNH			4.24 (0.56, 32.33)	3.94 (0.52, 29.93)	
SNC	1.66 (0.22, 12.63)	*2.53* (*0.60*, *10.87*)	*2.43* (*0.56*, *10.47*)	*3.34* (*0.99*, *11.24*)	≤*0.05*
Neuroblastoma	3.32 (0.43, 25.39)		2.37 (0.31, 17.92)	2.14 (0.28, 16.05)	

By diagnostic group and type of crop. The last column is the trend *p* value (Trend PV). ORs statistically significant are in italics. Results for the North Regions

**Table 4:** ORs by quartiles with reference group: 0 % in the Global Crop Index

Crop Global Index	1Q (0–2.55]	2Q (2.55–8.91]	3Q (8.91–26.42]	4Q (26.42–100]	Trend PV
Leukemia	0.37 (0.05, 2.71)	*2.64* (*1.42*, *4.91*)	*2.40* (*1.18*, *4.88*)	*3.91* (*1.68*, *9.08*)	≤*0.05*
HL		2.71 (0.36, 20.38)	*13.42* (*4.46*, *40.39*)		≤*0.05*
NHL		*3.95* (*1.40*, *11.17*)	*7.29* (*3.02*, *17.59*)		≤*0.05*
CNS	2.50 (0.76, 8.16)	0.49 (0.07, 3.55)	1.84 (0.57,5.96)	2.71 (0.63,11.61)	
Neuroblastoma	1.21 (0.16, 8.92)	1.43 (0.35, 5.93)	*3.51* (*1.25*, *9.88*)	*5.76* (*1.69*, *19.58*)	≤*0.05*
Retinoblastoma		1.74 (0.23,12.99)		4.45 (0.57,35.07)	
Renal tumors	2.60 (0.35, 19.49)	3.01 (0.71,12.71)	*7.30* (*2.53*, *21.07*)		≤*0.05*
Hepatic tumors	7.20 (0.91, 57.14)		5.97 (0.75, 47.71)	*11.61* (*1.35*, *100.17*)	≤*0.05*
Malignant bone tumors	3.87 (0.50, 29.75)	2.33 (0.31, 17.70)			
Sarcomas	2.37 (0.32, 17.75)	2.84 (0.67, 12.00)	3.44 (0.81, 14.66)		
Germ cell tumors	4.15 (0.54, 31.61)	*5.00* (*1.16*, *21.61*)	3.22 (0.43, 24.34)		

By diagnostic group and type of crop. Statistically significant ORs are in italics. Results for the Madrid Region

The results of the logistic regression models for the continuous variable are shown in Table 5. For the North regions the estimated ORs associated with an increment of 1 % in the total cultivated area showed an increased risk for all the individual causes, and only for retinoblastoma did the 95 % CI of OR include the value 1. These ORs ranged from 1.01 for leukemia to 1.03 for the malignant bone tumors (1 % in exposure area is equal to 3.14 hectares). Looking at the ORs by individual crops we observed that for irrigated crops, the statistically significant estimated ORs ranged from 1.01 for leukemia to 1.03 for hepatic tumors (1.03 germ cell), and for heterogeneous crops from 1.01 for leukemia to 1.02 for NHL and renal tumors. For fruit crops the statistically significant estimated ORs ranged from 1.02 for leukemia to 1.03 for malignant bone tumors. For vineyards only 2 cancer subtypes showed excess risks, hepatic tumors (OR = 1.03) and malignant bone tumors (OR = 1.03). And for olive crops only four cancer subtypes were exposed and 3 of them showed statistically significant excess risks of leukemia (OR = 1.02), CNS (OR = 1.03) and sarcomas (OR = 1.04). By cancer, CNS tumors showed statistically significant excess risks for all the analyses by individual crops: leukemia and sarcomas showed statistically significant excess risks for all crops but vineyards; and HL, NHL, neuroblastoma, bone and germ cells showed statistically significant excess risks for all crops but vineyards and olives.

**Table 5:** ORs and 95 % CIs associated with 1 % increment in the crop index by diagnostic group and type of crop for the North Region

Diagnostic group	Global Crop Index	Irrigated	Heterogeneous	Fruits	Vineyadrs	Olives
Leukemia	1.01 (1.01, 1.02)	1.01 (1.01, 1.02)	1.01 (1.01, 1.02)	1.00 (0.99, 1.02)	1.00 (0.99, 1.02)	1.02 (0.99, 1.05)
HL	1.02 (1.01, 1.03)	1.02 (1.00, 1.05)	1.02 (0.99, 1.05)	1.01 (0.98, 1.05)	1.01 (0.97, 1.06)	
NHL	1.02 (1.01, 1.03)	1.02 (1.01, 1.07)	1.02 (1.01, 1.04)	1.02 (1.01, 1.04)	1.00 (0.96, 1.04)	1.03 (0.99, 1.07)
CNS	1.02 (1.01, 1.02)	1.02 (1.01, 1.03)	1.01 (1.01, 1.02)	1.02 (1.01, 1.03)	1.01 (0.99, 1.03)	1.03 (1.00, 1.05)
Neuroblastoma	1.02 (1.01, 1.03)	1.02 (1.01, 1.03)	1.01 (1.00, 1.02)	1.02 (1.01, 1.03)	1.01 (0.99, 1.03)	1.01 (0.96, 1.06)
Retinoblastoma	1.01 (0.98, 1.02)	1.01 (0.98, 1.03)	1.00 (0.98, 1.03)	0.95 (0.82, 1.09)	0.99 (0.92, 1.06)	0.85 (0.33, 2.16)
Renal tumors	1.02 (1.01, 1.07)	1.019 (1.01, 1.03)	1.02 (1.01, 1.04)	0.99 (0.95, 1.04)	0.99 (0.94, 1.05)	
Hepatic tumors	1.02 (1.01, 1.04)	1.030 (1.01, 1.05)	1.00 (0.97, 1.04)	0.99 (0.92, 1.07)	1.03 (1.01, 1.06)	
Malignant bone t	1.02 (1.01, 1.04)	1.025 (1.01, 1.04)	1.02 (1.00, 1.04)	1.03 (1.01, 1.05)	1.03 (1.01, 1.05)	
Sarcomas	1.02 (1.08, 1.03)	1.021 (1.01, 1.03)	1.01 (0.99, 1.03)	1.01 (0.99, 1.03)	0.99 (0.94, 1.05)	1.04 (1.02, 1.07)
Germ cell tumors	1.02 (1.01, 1.04)	1.03 (1.02, 1.05)	1.02 (1.00, 1.03)	1.02 (0.99, 1.04)	1.01 (0.98, 1.05)	1.02 (0.97, 1.08)

For Madrid only irrigated and heterogeneous crops cover a relevant surface. The results for the continuous variables are shown in Table 6. The majority of the estimated ORs for Madrid were larger than those obtained for the North regions. For the Global Crop Index, leukemia (OR = 1.03), HL (OR = 1.04), neuroblastoma (OR = 1.07), renal (OR = 1.03) and hepatic tumors (OR = 1.05) showed statistically significant increased risks. Only six causes were exposed to irrigated crops and five of them showed statistically significant increased risks: leukemia with the lowest estimated OR, 1.02, and hepatic with the highest, 1.06. Also six causes were exposed to heterogeneous crops and two of them showed statistically significant increased risks of leukemia (OR = 1.05) and NHL (OR = 1.05).

**Table 6:** OR and 95 % CI associated with 1 % increment in the crop index by diagnostic group and type of crop for the Madrid Region

Diagnostic group	Global Crop Index	Irrigated	Heterogeneous
Leukemia	1.03 (1.02, 1.05)	1.02 (0.99, 1.05)	1.05 (1.03, 1.08)
HL	1.05 (1.01, 1.08)	1.05 (1.01, 1.09)	1.02 (0.91, 1.14)
NHL	1.03 (0.99, 1.06)	1.01 (0.95, 1.08)	1.05 (1.01, 1.09)
CNS	1.02 (0.99, 1.05)	1.03 (0.99, 1.06)	1.03 (0.99, 1.08)
Neuroblastoma	1.04 (1.01, 1.06)	1.04 (1.01, 1.07)	1.03 (0.98, 1.08)
Retinoblastoma	1.02 (0.98, 1.07)	1.04 (0.99, 1.09)	
Renal tumors	1.03 (1.00, 1.07)	1.04 (1.00, 1.08)	1.02 (0.93, 1.12)
Hepatic tumors	1.05 (1.01, 1.09)	1.06 (1.01, 1.10)	1.04 (0.95, 1.13)
Malignant bone t.	0.96 (0.80, 1.15)		1.01 (0.87, 1.16)
Sarcomas	1.08 (0.97, 1.06)	0.99 (0.89, 1.11)	1.04 (0.99, 1.10)
Germ cell tumors	1.08 (0.95, 1.08)		1.04 (0.98, 1.11)

## Discussion

In this study, we investigated the effects on childhood cancer risk of exposure to crops that are generally treated with pesticides, taking into account different types of crops. Our findings support the hypothesis that living near crops might be a risk factor for childhood malignant tumors. Certainly, our analyses show an excess of risk of childhood cancer among children living in the proximity of crops. In view of the results, we think that the proposed crop index, Global Crop Index, is a good approach to evaluate the exposure to pesticides and its possible association with childhood cancer. Our results showed an excess of risk with leukemia and showed no association with retinoblastoma. For leukemia there are many studies that associate pesticide exposure with leukemia risk [7, 11]. On the other hand, we did not find an association with retinoblastoma, which is a tumor with an inherited component in 40 % of the cases [26]. These results show the potential accuracy of the proposed crop index. With regard to exposure to pesticides, the existing literature about childhood cancer shows that there is evidence of some association between pesticide exposure and cancer in children. The reviews from Zahm and Ward in 1998, Infante-Rivard and Weichenthal in 2007, Vinson in 2011, and Chen in 2015 [7, 9–11] went over dozens of papers published since the late 70 s. Most of these studies were case–control and cohort studies evaluating parental exposure, occupational exposure, residence on a farm, or household use, to or of pesticides at different timings of exposure: pre-conception, during pregnancy and in childhood. For the discussion and comparison of our results with previous work we will to refer to these useful review articles.

In relation to the best addressed diagnostic group, leukemia, many studies have suggested an association between exposure to pesticides and these cancers originating in the bone marrow. According to these reviews, there is an association between pesticide exposure and childhood leukemia [7, 9–11]. Specifically, exposure to household insecticides and parental exposure before and during pregnancy could increase the risk for childhood leukemia. Regarding NHL, despite the limited number of studies, it seems that pesticides play a role in the development of these tumors. Some studies included in these reviews also reported a gradient in the response to increasing exposure; as in these studies, our results suggest increasing risk with increasing level of exposure [7, 11]. For CNS tumors, the literature tends to support the position that exposure to pesticides could be associated with brain cancer [7–11]. Some studies suggest that the greatest risks are associated with household insecticide use and prenatal exposure to insecticides [7]. Regarding neuroblastoma the number of studies is limited; however, these studies suggest a potential relationship between occupational pesticide exposure of the parents and neuroblastoma in children [7, 11]. For other childhood cancers available information is scant. For renal tumors the studies suggest that parental exposure before birth increases the risk. And for malignant bone tumors the findings suggest that occupational pesticide exposure of the parents at conception or during pregnancy were associated with increased risk [7, 11]. In our study we were unable to evaluate parental exposure, household use or the timing of exposure at an individual level, nor evaluate exposure of the mother during pregnancy or after birth. However, our results for every individual diagnostic group point to risk increments such as those in the aforementioned studies. The comparison of our results with the results from the previous studies is complicated because the exposure variables are very different. The study which most closely approximates ours is that on agricultural crop density from Booth et al. [27]. They estimated the relative risk (RR) of childhood cancer associated with an increase of 1 % in the crop density at county level; i.e. for total leukemia cases they reported a RR = 1.09 associated with dried bean crops and a RR = 1.11 associated with sugar beet.

CORINE Land Cover is a good tool to study environmental variables. As yet, it is the most complete database of land use in Europe [23]. As already noted, we used the subcategories of the agricultural class, but we should mention that there are two additional subcategories: non-irrigated arable land and pastures; we did not include these in the analysis. We decided to exclude them because of the Spanish law (Real Decreto 1311/2012 [28]) regarding sustainable land use and the use of pesticides. Under this regulation, non-irrigated arable land and pastures are classified as areas exposed to very low pesticide doses [29]. A potential problem is that the CORINE Land Cover database does not show changes over time: it was done for the year 2006. Nevertheless, that should not be a significant issue in our study because that year is included within the studied period. However, CORINE Land Cover used satellite images to validate land use. The validation was based on the reinterpretation of field photographs and the original satellite images [23].

Land use/cover databases have been employed in different studies to measure the exposure to pesticides. For a study conducted in Texas, USA, the authors used aerial photographs and digital maps to identify agricultural fields proximate to birth residence [4]. They defined a buffer of 1000 meters around the residence and found associations with only a few cancers. For that study there were 1190 cases and 2059 controls, while for our study we had 3350 cases and 20,365 controls—which increase the statistical power of the analysis significantly. In two other American studies, a land cover database was employed to build a variable about exposure to crops in order to study the association between crop exposure crops and risk of lymphohematopoietic cancer in a women´s cohort [30, 31]. In these studies, the authors utilized distances to crops between 250 and 1000 m. For our study, we took a maximum distance of 1000 m as exposure area. We decided to use a larger distance due to the extent of the studied area, which included several regions and many different landscapes that could have affected the dispersion of the pesticides. We also calculated the density of crops within the exposure area; some previous studies have also employed this approach [27, 32]. A recent study in the USA utilized crop density at county level to study the risk of childhood cancer, showing increases in risk for leukemia and CNS tumors [27].

One of the limitations of this study is the non-inclusion of individual data about possible confounding factors that might be associated with the distance, as socioeconomic variables or life-style-related factors. As we did not have data for individuals, we used socioeconomic data at census track level to include some socioeconomic information in the analysis. Another limitation could be the use of a circular buffer around the home residence as a proxy of exposure, assuming an isotropic model, something that could introduce a problem of misclassification, since real exposure is critically dependent on prevailing winds, geographic landforms and releases into aquifers. Nevertheless, this problem would limit the capacity to find positive results but in no way invalidates the associations found. An additional important limitation is that we did not have any information about occupational exposure of the parents at an individual level, which seems to be one of the identifiable risk factors. Lastly, we did not have information about the specific pesticides used in crops in Spain. However, as mentioned previously, we chose the agricultural uses in CORINE Land Cover that have legal regulations for pesticide treatments.

It should be noted that we have the home addresses of the cases at the moment of diagnosis and, for the controls, the home address of the mother at birth. This difference could introduce bias in the analysis but, according to official data, in Spain only around 1 % of the child population change residence to a different province [24]. Therefore, we considered that the home address at diagnosis is the same as the home address at birth for the majority of the cases.

On the other hand, one of the main strengths of our study is the large control group. Most studies of this type have one or two controls per case [33–35]. In our study we have 6 controls per case and that gives a much more realistic image of the spatial distribution of the population at risk. A further advantage of the study is the stratification of the risk by type of crop, which provides a more exhaustive description of childhood cancer risk.

## Conclusion

Despite of the limitations of this study, our result points to the same conclusion as many previous studies and suggests that living in the proximity of cultivated land could be associated with many types of cancer in children. However, these findings need to be replicated in other studies with detailed information on individual level exposures for children and parents, pesticide use data and other potentially confounding factors. Furthermore, this study shows how land use information, which is publicly available for many countries, can be used to approximate exposure to crops.

## Abbreviations

GIS: geographical information system
HL: Hodgkin’s lymphoma
NHL: non-Hodgkin’s lymphoma
RETI-SEHOP: Spanish Registry of Childhood Tumors
CNS: central nervous system neoplasms
INE: Instituto Nacional de Estadística
Irrigated: irrigated land
Rice: rice fields
Fruits: fruit trees and berry plantations
Olives: olive groves
Heterogeneous: heterogeneous agricultural areas

## References

[1] Peris-Bonet R, Salmeron D, Martinez-Beneito MA, Galceran J, Marcos-Gragera R, Felipe S (2010). Childhood cancer incidence and survival in Spain. Ann Oncol.

[2] Instituto Nacional de Estadística. Defunciones según la Causa de Muerte 2012 (Internet). 2015 (cited 2015 Jan 11). http://www.ine.es/jaxi/tabla.do?path=/t15/p417/a2012/l0/&file=01001.px&type=pcaxis&L=0.

[3] Peris-Bonet R, Ruiz Martinez N, Felipe Garcia S, Pardo Romaguera E, Valero Poveda S. Cáncer infantil en España. Estadísticas 1980–2012. Registro Nacional de Tumores Infantiles (RNTI-SEHOP). Valencia: Universitat de València; 2013.

[4] Carozza SE, Li B, Wang Q, Horel S, Cooper S (2009). Agricultural pesticides and risk of childhood cancers. Int J Hyg Environ Health.

[5] Flower KB, Hoppin JA, Lynch CF, Blair A, Knott C, Shore DL (2004). Cancer risk and parental pesticide application in children of Agricultural Health Study participants. Environ Health Perspect.

[6] Garry VF (2004). Pesticides and children. Toxicol Appl Pharmacol.

[7] Infante-Rivard C, Weichenthal S (2007). Pesticides and childhood cancer: an update of Zahm and Ward’s 1998 review. J Toxicol Environ Health B Crit Rev.

[8] Jurewicz J, Hanke W (2006). Exposure to pesticides and childhood cancer risk: has there been any progress in epidemiological studies?. Int J Occup Med Environ Health.

[9] Chen M, Chang CH, Tao L, Lu C (2015). Residential exposure to pesticide during childhood and childhood cancers: a meta-analysis. Pediatrics.

[10] Vinson F, Merhi M, Baldi I, Raynal H, Gamet-Payrastre L (2011). Exposure to pesticides and risk of childhood cancer: a meta-analysis of recent epidemiological studies. Occup Environ Med.

[11] Zahm SH, Ward MH (1998). Pesticides and childhood cancer. Environ Health Perspect.

[12] Daniels JL, Olshan AF, Savitz DA (1997). Pesticides and childhood cancers. Environ Health Perspect.

[13] Allpress JL, Curry RJ, Hanchette CL, Phillips MJ, Wilcosky TC (2008). A GIS-based method for household recruitment in a prospective pesticide exposure study. Int J Health Geogr.

[14] Lu C, Fenske RA, Simcox NJ, Kalman D (2000). Pesticide exposure of children in an agricultural community: evidence of household proximity to farmland and take home exposure pathways. Environ Res.

[15] Ward MH, Nuckols JR, Weigel SJ, Maxwell SK, Cantor KP, Miller RS (2000). Identifying populations potentially exposed to agricultural pesticides using remote sensing and a geographic information system. EnvironHealth Perspect.

[16] Ward MH, Lubin J, Giglierano J, Colt JS, Wolter C, Bekiroglu N (2006). Proximity to crops and residential exposure to agricultural herbicides in iowa. Environ Health Perspect.

[17] Xiang H, Nuckols JR, Stallones L (2000). A geographic information assessment of birth weight and crop production patterns around mother’s residence. Environ Res.

[18] López-Abente G, Pollán M, Ardanaz E, Errezola M (2003). Geographical pattern of brain cancer incidence in the Navarre and Basque Country regions of Spain. Occup Environ Med.

[19] Chang ET, Adami HO, Bailey WH, Boffetta P, Krieger RI, Moolgavkar SH (2014). Validity of geographically modeled environmental exposure estimates. Crit Rev Toxicol.

[20] Garcia-Perez J, Lopez-Abente G, Gomez-Barroso D, Morales-Piga A, Romaguera EP, Tamayo I (2015). Childhood leukemia and residential proximity to industrial and urban sites. Environ Res.

[21] Ramis R, Gomez-Barroso D, Tamayo I, Garcia-Perez J, Morales A, Pardo RE (2015). Spatial analysis of childhood cancer: a case/control study. PLoS ONE.

[22] Steliarova-Foucher E, Stiller C, Lacour B, Kaatsch P (2005). International classification of childhood cancer, third edition. Cancer.

[23] European Environment Agency EEA. CORINE land cover 2006 (Internet). 2015 (cited 2015 Jan 11). http://www.eea.europa.eu/publications/COR0-landcover.

[24] Instituto Nacional de Estadística. Censos de Población y Viviendas de 2001. http://www.ine.es/censo2001/. 2015.

[25] The Comprehensive R Archive Network. The comprehensive R archive network (Internet). 2015. http://cran.r-project.org/.

[26] Cavenee WK, Dryja TP, Phillips RA, Benedict WF, Godbout R, Gallie BL (1983). Expression of recessive alleles by chromosomal mechanisms in retinoblastoma. Nature.

[27] Booth BJ, Ward MH, Turyk ME, Stayner LT (2015). Agricultural crop density and risk of childhood cancer in the midwestern United States: an ecologic study. Environ Health.

[28] B. O. E. Real Decreto 1311/2012. https://www.boe.es/boe/dias/2012/09/15/pdfs/BOE-A-2012-11605.pdf. 2015.

[29] Ministerio de Agricultura A y MAG de E. Requisitos que ha de cumplir la documentación de asesoramiento en el ámbito de la producción agraria. http://www.magrama.gob.es/es/agricultura/temas/sanidad-vegetal/DOCUMENTACION_DE_ASESORAMIENTO_tcm7-289003.pdf. 2015.

[30] Jones RR, Yu CL, Nuckols JR, Cerhan JR, Airola M, Ross JA (2014). Farm residence and lymphohematopoietic cancers in the Iowa Women’s Health Study. Environ Res.

[31] Jones RR, DellaValle CT, Flory AR, Nordan A, Hoppin JA, Hofmann JN (2014). Accuracy of residential geocoding in the Agricultural Health Study. Int J Health Geogr.

[32] Pollan M, Lopez-Abente G, Aragones N, Ruiz M (1999). Malignant brain tumour mortality among children and adolescents: geographical distribution in Spain. J Neurol Sci.

[33] Badaloni C, Ranucci A, Cesaroni G, Zanini G, Vienneau D, Al-Aidrous F (2013). Air pollution and childhood leukaemia: a nationwide case–control study in Italy. Occup Environ Med.

[34] Pedersen C, Raaschou-Nielsen O, Rod NH, Frei P, Poulsen AH, Johansen C (2014). Distance from residence to power line and risk of childhood leukemia: a population-based case–control study in Denmark. Cancer Causes Control.

[35] Selvin S, Ragland KE, Chien EY, Buffler PA (2004). Spatial analysis of childhood leukemia in a case/control study. Int J Hyg Environ Health.

